# MBT3D: Deep learning based multi-object tracker for bumblebee 3D flight path estimation

**DOI:** 10.1371/journal.pone.0291415

**Published:** 2023-09-22

**Authors:** Luc Nicolas Stiemer, Andreas Thoma, Carsten Braun

**Affiliations:** 1 Department of Aerospace Engineering, FH Aachen, Aachen, North Rhine-Westphalia, Germany; 2 Department of Aerospace Engineering, RMIT University, Melbourne, Victoria, Australia; National University of Sciences and Technology NUST, PAKISTAN

## Abstract

This work presents the Multi-Bees-Tracker (MBT3D) algorithm, a Python framework implementing a deep association tracker for Tracking-By-Detection, to address the challenging task of tracking flight paths of bumblebees in a social group. While tracking algorithms for bumblebees exist, they often come with intensive restrictions, such as the need for sufficient lighting, high contrast between the animal and background, absence of occlusion, significant user input, etc. Tracking flight paths of bumblebees in a social group is challenging. They suddenly adjust movements and change their appearance during different wing beat states while exhibiting significant similarities in their individual appearance. The MBT3D tracker, developed in this research, is an adaptation of an existing ant tracking algorithm for bumblebee tracking. It incorporates an offline trained appearance descriptor along with a Kalman Filter for appearance and motion matching. Different detector architectures for upstream detections (You Only Look Once (YOLOv5), Faster Region Proposal Convolutional Neural Network (Faster R-CNN), and RetinaNet) are investigated in a comparative study to optimize performance. The detection models were trained on a dataset containing 11359 labeled bumblebee images. YOLOv5 reaches an Average Precision of *AP* = 53, 8%, Faster R-CNN achieves *AP* = 45, 3% and RetinaNet *AP* = 38, 4% on the bumblebee validation dataset, which consists of 1323 labeled bumblebee images. The tracker’s appearance model is trained on 144 samples. The tracker (with Faster R-CNN detections) reaches a Multiple Object Tracking Accuracy *MOTA* = 93, 5% and a Multiple Object Tracking Precision *MOTP* = 75, 6% on a validation dataset containing 2000 images, competing with state-of-the-art computer vision methods. The framework allows reliable tracking of different bumblebees in the same video stream with rarely occurring identity switches (*IDS*). MBT3D has much lower *IDS* than other commonly used algorithms, with one of the lowest false positive rates, competing with state-of-the-art animal tracking algorithms. The developed framework reconstructs the 3-dimensional (3D) flight paths of the bumblebees by triangulation. It also handles and compares two alternative stereo camera pairs if desired.

## Introduction

It is in the nature of the buff-tailed bumblebee (*Bombus terrestris*) to fly efficiently in cluttered environments [1]. Due to their sophisticated spatial behavior, they quickly determine and maintain the most suitable routes among flowers and other obstacles due to their sophisticated spatial behavior [2]. They solve complex navigational tasks in their daily live [3]. Currently, industry demand for autonomously operating aerial vehicles is steadily increasing, also the need for reliable collision avoidance algorithms increases. Unmanned Aerial Vehicles (UAV) operating in urban environments face similar challenges as bumblebees in their daily life. Thus, *Bombus terrestris* is a perfect fit as a bionic model for UAV collision avoidance enhancements. Therefore, a highly detailed investigation of the behavior of *Bombus terrestris* will benefit the research in bio-inspired obstacle avoidance for UAV. This study presents a *Bombus terrestris* tracking implementation, including reconstruction of the 3-dimensional (3D) flight paths.

Trackers have been commonly used in biology to monitor and track the behavior of animals. Several different methods were developed, with specific advantages and disadvantages. Many trackers and detectors have specific requirements on the video for reliable performance. Often sufficient lighting, high contrast in color or intensity between animals and background, lack of occlusion and reflections, continuously visible animals, and stable conditions within trials, across trials and days are required [4]. However, the necessities of the experimental setup for video analysis sometimes influence the result (e.g., [5]). Therefore, the lower the requirements on the analysis software, the easier the focus is on the experiment itself. Additionally, highly reliable and robust analysis systems allow tracking in an animal’s natural environment and not only in a clean laboratory setup. To the best of the author’s knowledge tracking multiple interacting and visually indistinguishable animals is still a challenging, unsolved problem in Computer Vision [4].

The continuous improvement of deep learning algorithms results in significant advancements within Computer Vision problems such as Multi-Object Tracking (MOT) [6]. MOT possesses demanding tasks such as maintaining the identities of objects of interest, handling long-time occlusions, motion predictions, and re-identifications. Further, tracking insects in social groups is exceptionally challenging due to their tendency for intensive interactions and occlusions between multiple individuals, their similarity in appearance, sudden movement changes [7] and their small size [8]. Most state-of-the-art MOT methods address these problems by relying on Tracking-by-Detection, requiring a sound performance of the upstream detector. These methods have the advantage of simplifying the problem into two sub-problems: The detection of the objects of interest, which focuses on classifying and locating objects of interest in each frame independently, and the more challenging task of associating these detections to already existing trajectories. Motion prediction or appearance prediction models usually perform data. Most recent studies in Computer Vision focus on pedestrian tracking [9], as industrial interest is exceptionally high here, e.g., collision avoidance for autonomous driving or surveillance purposes. These trackers often use Social Force Models for motion prediction [10, 11]. However, constant velocity assumptions are also used widely [12]. An exception among pedestrian trackers is the Tracking without bells and whistles (Tracktor++) algorithm [13], defining new state-of-the-art performance in three Multi-Object Tracking benchmarks in the MOT Challenge [14]. Tracktor++ completely avoids motion models and performs data association only by exploiting the regression head of a detector model. Regression heads in the classical sense predict the localization of the object bounding box, but Tracktor++ exploits them further to perform temporal realignment of the bounding boxes. This strategy is very efficient for pedestrian tracking because pedestrian identities have large differences in appearance (e.g., clothing, bags, hair), and thus, introducing a motion model improves the association hardly [13]. However, in insect tracking, the identities are very similar in appearance, so it is difficult to solve the association problem without using a motion model [7]. Nonetheless, biologists have been tracking insects for several decades. Initially, tracking was performed manually (e.g., [15]). Later, tracking was supported by various tools, e.g., for 3D reconstruction from two camera videos [16]. Investigation of multiple animals is often subdivided into several tasks. [17] formulate three linear assignment problems (2D tracks with constant identity, matching similar identities in multiple videos, linking 3D track segments by global optimization). [18] also follows a global optimization scheme with a recursive divide and conquer approach, which uses information from the alternate video to handle occlusion. Several studies published Tracking methods focusing on Insect Tracking [19], or more specifically, Bumblebee Tracking, e.g., [20, 21] in the years 2012 and 2014.

In recent years, [22] introduced a three-dimensional tracking method for small insects. However, their approach relies on background extraction for detection, which restricts its practicality to controlled laboratory environments. Another approach, presented in [23], utilizes a Tracking-By-Detection method for real-time insect tracking. The authors of [23] draw inspiration from the tracking method outlined in [24]. Their primary objective was to count relatively stationary insects, and they achieved this by assuming that the shortest distance between consecutive frame detection candidates corresponds to the same insect identity. While this simplification sufficed for their purposes, it falls short for our specific goal of tracking flying bumblebees that frequently occlude each other. Alternatively, TrichTrack [25] offers another intriguing Tracking-By-Detection method, featuring a linear velocity model for motion matching and a reidentification model for appearance matching. This combination holds promise for accurately tracking flying bumblebees. Unfortunately, the source code for TrichTrack is no longer accessible, which limits its immediate applicability.

[7] developed a Multi-Ant Tracker, showing promising results on MOT benchmarks. This method performs data association by two branches, an appearance matching model and a motion matching algorithm. The first branch consists of an offline trained ResNet model with 128-dimensional feature vectors for appearance description. Each trajectory is allocated to a set of descriptors **K**_*i*_ of the 100 most recent images. The matching degree *d*^(1)^(*i*, *j*) between the *ith* trajectory and the *jth* detection is computed as:

$$\begin{array}{c} d^{\left(1\right)}\left(i,j\right)=\underset{k}{\text{min}}\{1-r_{j}r_{k}^{\left(i\right)}\}for\;r_{k}^{\left(i\right)}\in K_{i} \end{array}$$ (1)

where *r*_*k*_ defines the *kth* appearance descriptor of the *ith* trajectory [7] and *r*_*j*_ denotes the eigenvector value of the *jth* detection according to the cosine similarity association model [7]. If the matching degree *d*^(1)^(*i*, *j*) is higher than a pre-defined threshold, the *jth* detection is potentially related to the *ith* trajectory.

The second branch, the motion matcher, is based on a Kalman Filter with a constant velocity motion and observation model [26]. The Kalman Filter gets two inputs: the observation models prediction, the *jth* detection, and the target state prediction of the *ith* trajectory estimated by a constant velocity motion model. The Kalman Filter corrects these two state predictions to Kalman states. The square of the Mahalanobis distances between predicted Kalman states and detections is then calculated as follows:

$$\begin{array}{c} d^{\left(2\right)}\left(i,j\right)={\left(d_{j}-y_{j}\right)}^{T}S_{i}^{-1}\left(d_{j}-y_{i}\right) \end{array}$$ (2)

where **d**_*j*_ depicts the bounding box of the *jth′s* detection, and **y**_*i*_ represents the Kalman state of the *ith* trajectory. **S**_*i*_ denotes the covariance matrix of the Kalman Filter. If a specific threshold is reached, *d*^(2)^(*i*, *j*) indicates a potentially successful association [7].

Finally, the matrices $D^{\left(1\right)}=d_{i,j}^{\left(1\right)}$ and $D^{\left(2\right)}=d_{i,j}^{2)}$ solve the association problem. In other words, they assign each detection to a trajectory [26]. This method provides robust behavior for short-term predictions by exploiting object motion information. Further, it reliably recovers from long-term occlusion by cosine distance of appearances. Thus, it defines new state-of-the-art MOT benchmarks and is highly robust regarding insect tracking problems. This method is implemented in the proposed framework to perform the data association task of the Tracking-By-Detection paradigm [7].

A robust detection model is indispensable to provide reliable detections for the data association task. In the last decades, two different detector architectures have gained acceptance: One-staged and two-staged detectors. One-staged detectors focus on the speed, therefore, trading-off accuracy. Thus, they are usually outperformed in localization and object recognition accuracy by two-staged networks. Two-staged detectors simplify the detection problem by dividing it into two sub-tasks, the proposal of regions and the classification [27].

The Faster Region Proposal Convolutional Neural Network (Faster R-CNN) [28] is currently the most representative two-stage detector [29, 30]. It consists of two branches, a Fully Convolutional Network for region proposal (RPN) and a detector model which uses these region proposals as input. Both branches share the same backbone network for feature extraction. The first branch, the RPN, takes 3*x*3 sliding windows of these feature maps as input, applying *k* anchor boxes on each sliding window. These anchor boxes are the input of a convolutional layer. The output feeds two parallel branches, one for classification and one for region regression. The classification branch returns probabilities on whether an anchor box is considered foreground (object of interest) or background (no object of interest). The regression branch predicts offsets from the anchor boxes to create the final proposals [28, 31]. The second branch, the detector model, consists of a Region of Interest (RoI) Pooling layer and three fully connected layers. It takes in the region proposals and converts them to a fixed size exploiting RoI Pooling. The fixed-size proposals feed the fully connected layers, which perform classification for confidence score determination and regression for bounding box estimation. Predicting an offset between region proposals and ground truth bounding boxes gives the regression [31]. Training the two branches of the network with a shared backbone model is challenging. Therefore, [31] introduces a 4-step training algorithm.

The *first step* is training the RPN by backpropagation and stochastic gradient descent. A per image loss function is defined as follows:

$$\begin{array}{cc} L(\left\{\hat{p}\right\}\left\{p\right\}\left\{\hat{t}\right\}\left\{t\right\}) & =\frac{1}{N_{cls}}\sum_{n=i}Loss_{cls}(\hat{p_{i}},p_{i})\\ & +\lambda \frac{1}{N_{reg}}\sum_{n=i}p_{i}Loss_{reg}(\hat{t_{i}},t_{i}) \end{array}$$ (3)

where $\hat{p_{i}}$ is the predicted probability of the *ith* anchor being foreground or background. *p*_*i*_ is the corresponding ground-truth label, either 1 for foreground or 0 for background. The *Loss*_*cls*_ is a cross-entropy loss between the prediction $\hat{p_{i}}$ and the ground truth *p*_*i*_. ${\hat{t}}_{i}$ is a vector representing the coordinates of the predicted bounding box, and *t*_*i*_ is equivalently a vector of the ground-truth bounding box coordinates. For the regression loss, [31] use $Loss_{reg}({\hat{t}}_{i},t_{i})=R({\hat{t}}_{i},t_{i})$ where *R* is the smooth *L*1 loss defined in [31]. Furthermore, the two terms are balanced by λ and normalized by *N*_*cls*_ and *N*_*reg*_ [28]. The weights of the RPN are initialized by extracting them randomly from a zero-mean Gaussian distribution with a standard deviation of 0.01, while the weights of the backbone are initialized by using the weights of an “ImageNet-pretrained model” [28].

During the *second step*, the detection model is trained by receiving the region proposals of the RPN with an ImageNet pre-trained model [28] for finetuning on the detection task. At this point, the RPN and the Faster R-CNN detector do not share the same backbone layers. In the *third step*, the detector’s backbone initializes a second RPN training. Here, the detector’s backbone layers are frozen, i.e., fixing their weights during training. Thus, only the unique RPN layers are trained during the third step.

During the *fourth step*, the unique layers of the detector are finetuned by freezing the shared backbone and the RPN.

Common one-stage methods are You Only Look Once (YOLO) [29] and RetinaNet [30]. RetinaNet exploits the faster principle of Feature Pyramid Networks (FPN) instead of using a Region Proposal Network like R-CNN. It consists of a ResNet backbone model as a bottom-up pathway and the FPN as a top-down pathway. The ResNet extracts backbone features from the image, and each convolutional layer of the ResNet passes its feature maps to a corresponding convolutional layer of the FPN. The FPN up-samples semantic rich feature maps by a nearest neighbor algorithm. After up-sampling, the features of the corresponding bottom-up layer add up elementwise to compensate for the loss of resolution information. This summation results in feature maps saturated in both semantics and resolution [30]. These saturated feature maps are passed to a classification head and a regression head. Based on a predefined set of anchors, the classification head predicts the probability of object presence of a specific class. The regression head predicts the offset between each anchor box and the ground truth of the bounding box. The authors [30] present a novel focal loss for training purposes. They observed that redirecting the focus of the standard cross-entropy loss towards poorly predicted samples significantly minimizes the loss, especially for class-imbalanced datasets. Summing up the loss over multiple easy-to-detect examples, small loss values will overwhelm rare classes and reduce their impact. Therefore, they define the focal loss for binary classification as:

$$\begin{array}{c} L_{focal}=-{\left(1-\hat{y_{t}}\right)}^{\gamma }\cdot log(\hat{y_{t}}) \end{array}$$ (4)

where *γ* represents the focal parameter and $\hat{y_{t}}$:

$$\hat{y_{t}}\left\{\begin{array}{ll} 1-\hat{y} & if\;y=1\\ \hat{y} & otherwise \end{array}\right.$$ (5)

$\hat{y}$ is the predicted probability of the ground truth class (between 0 and 1), and *y* is the ground truth class itself (either 0 or 1).

The second commonly used one-stage detector is the YOLO method. We utilize YOLOv5 [32] algorithm. YOLOv5 implements a Cross-stage local network (CSPDARKnet53) [33] as a backbone with an added Spatial Pyramid Pooling (SPP) block, increasing the receptive field and separating the most significant context features while maintaining high network speed operation [29]. A Path Aggregation Network (PANet) [34] forms the path-aggregation neck, and the YOLOv3 [35] head is reused. Major improvements of YOLOv5 includes auto anchor box optimization and mosaic data augmentation.

The X101-FPN is the best performing Faster R-CNN model, achieving an Average Precision of *AP* = 43% on the Common Objects in Content (COCO) Dataset (https://cocodataset.org). COCO is a large object detection dataset with 80 classes and more than 1.5 million object instances. For simplification, X101-FPN is abbreviated as Faster R-CNN.

The R101 RetinaNet is the best performing RetinaNet architecture, achieving an Average Precision of *AP* = 40.4% while being slightly faster than the R-CNN models.

The YOLOv5 models define new state-of-the-art benchmarks and outperform all existing detectors remarkably ([29]; https://github.com/ultralytics/yolov5). It introduces four different model types: YOLOv5s, YOLOv5m, YOLOv5l and YOLOv5x. Both YOLOv5s and YOLOv5m are suitable for tasks requiring fast computations, meanwhile YOLOv5l and YOLOv5x focus on precision. We chose YOLOv5l because high precision is required and YOLOv5x shows only slight precision improvements but has high computational effort. The YOLOv5l model, for instance, achieves *AP* = 48, 2% on the COCO dataset while being significantly faster than R-CNN or RetinaNet. Therefore, the proposed framework includes the YOLOv5l model, besides the Faster R-CNN and the RetinaNet.

In summary, the major contributions of this work are as follows:

- We propose the Multi-Bees-Tracker (MBT3D), a framework specifically designed for tracking flight paths of bumblebees in a social group. MBT3D enables accurate and reliable tracking of bumblebee flight paths, providing valuable insights into their behavior within social groups.
- We train an appearance matching model and several detection models on our bumblebee datasets.
- We evaluate the performance of the tracker compared to several tracking tools widely used in the biology community.
- We provide one dataset for detection training consisting of 11, 359 labeled images, one for appearance model training with 144 samples, and a separate dataset containing 2, 000 frames for evaluating tracking performance.

The rest of this paper is structured as follows. Section **Materials and methods** presents the proposed framework architecture, implementation, and dataset preparation details. Section **Results** discusses and evaluates the training of both detection and tracking networks. In the next Section, **Performance compared to other trackers** is evaluated before we come to the **Conclusion**.

$$\begin{array}{c} P_{Y}=\underset{S_{Y}}{\underset{︸}{H\left(Y_{n}\right)-H\left(Y_{n}|V_{n}^{Y}\right)}}+\underset{T_{X\to Y}}{\underset{︸}{H\left(Y_{n}|V_{n}^{Y}\right)-H\left(Y_{n}|V_{n}^{X,Y}\right)}}, \end{array}$$ (6)

## Materials and methods

### Hardware, software

The implementation and training of the deep learning models were executed on an Ubuntu 18.04 OS. The machine is equipped with an IntelCore i9–10900KF, 64 GB RAM and an Nvidia RTX 3090.

### MBT3D framework

**Fig 1:**
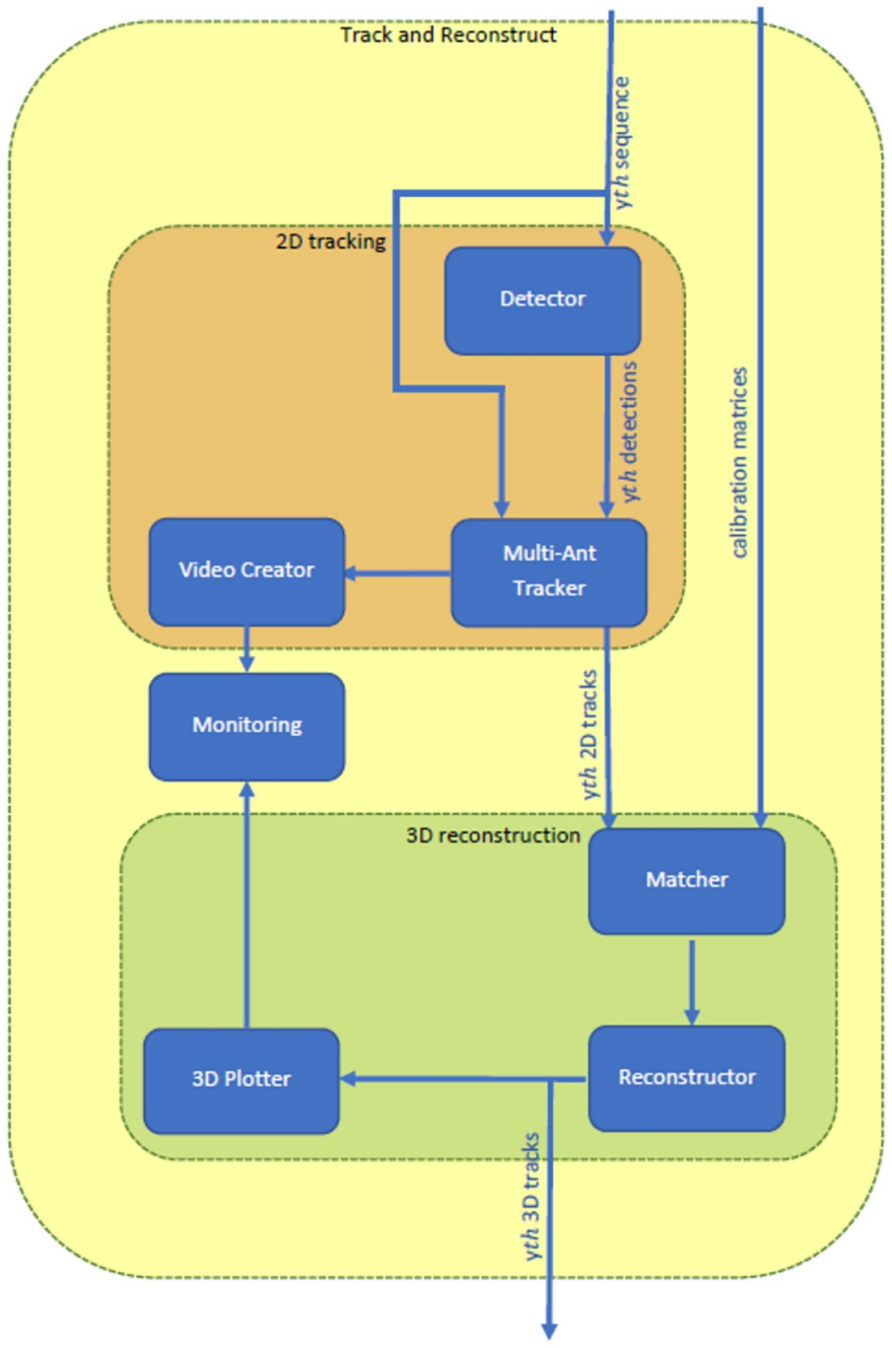
MBT3D architecture.

We propose a framework that combines the Multi-Ant Tracker for data association and an arbitrary detector model (e.g., YOLOv5, RetinaNet, or Faster R-CNN). The framework can handle and compare two alternative stereo camera pairs. Its architecture is shown in Fig 1.

It receives the calibration matrices and the images of the *yth* sequence of camera 1, 2, and 3 as input. It then performs detection on all images of each camera sequence and passes the detections to the Multi-Ant Tracker for 2D track prediction (Fig 1, orange box). The Multi-Ant Tracker receives the detections as input and outputs predicted bounding boxes, one for each tracked object with the corresponding identity number. The predicted bounding boxes and their identity numbers are drawn on the corresponding frame by the Video Creator and further displayed by the Monitoring script. Further, the predicted bounding boxes pass to the 3D reconstruction algorithm (Fig 1, green box).

**Fig 2:**
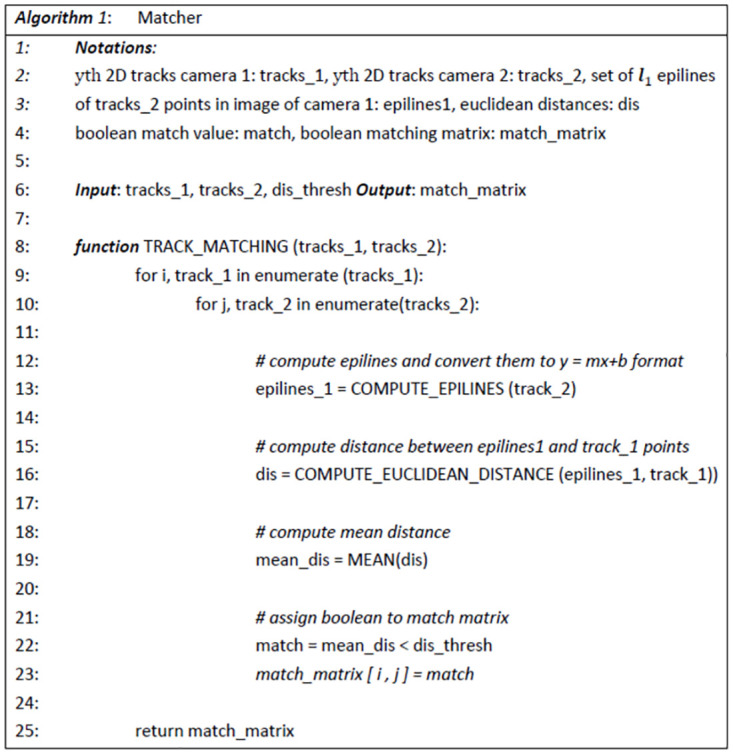
Matcher algorithm. The algorithm matches *yth* 2*D* tracks of camera 1 with the *yth* tracks of camera 2 and 3.

A classic triangulation algorithm performs the 3*D* reconstruction. The three cameras 1, 2, and 3 combine into the camera pairs 12 and 13 for triangulation. The *yth* 2*D* tracks of camera 1 have to match the *yth* tracks of camera 2 and 3 for triangulation. Therefore, we introduce a Matcher algorithm taking the *yth* 2*D* tracks and the calibration matrices as input and outputs a boolean *match*_*matrix*. The *match*_*matrix* expresses which track identity of camera 1 belongs to which track identity in camera 2, or 3, respectively. Fig 2 shows the matcher algorithm.

It compares each *yth* 2D tracks of camera 1 with each *yth* 2D tracks of camera 2 by computing the distances between epilines and image points. Assuming an image point *x*_2_ in camera 2, the corresponding point *x*_1_ in camera 1 lays on the epiline *l*_1_. If a track in camera 1 corresponds to a track in camera 2, the distances between *epilines*_1 (a set of *l*_1_) and points of *track*_1 (a set of *x*_1_) is close to 0. In line 13, the *epilines*_1 are calculated exploiting the formula:

$$\begin{array}{c} l_{1}=F\;x_{2} \end{array}$$ (7)

where **l**_1_ is an element of the *epilines*_1 array in the image of camera 1, corresponding to the point *x*_2_, which is an element of the *tracks*_2 array. The fundamental matrix between camera 1 and 2 is denoted as **F**. In line 16, the minimal distances between *epilines*_1 and *track*_1 points are calculated. In line 19, the mean distance between *epilines*_1 and *track*_1 is determined after removing outliers. In lines 22–23, the corresponding element of the *match*_*matrix* is assigned with a boolean. The boolean is True if the mean distance is below the given threshold (8 pixels appears to be an appropriate threshold, determined empirically) and False if the mean distance is above the given threshold; leading to the *match*_*matrix*:

$$\begin{array}{c} match\_matrix=[\begin{array}{ccc} match_{11} & \ldots & match_{1j}\\ \vdots & \ddots & \vdots \\ match_{i1} & \ldots & match_{ij} \end{array}] \end{array}$$ (8)

where *match*_*ij*_ is the boolean match of the *ith* identity of *tracks*_1 and the *jth* identity of *tracks*_2.

The Matcher passes the *match*_*matrix* to the Reconstructor, which performs 3D reconstruction by triangulation for the matched tracks (green box in Fig 1). It passes the 3D tracks to the 3D plotter. The interactive 3D plot is displayed in HyperText Markup Language (HTML) format by the Monitoring script. 2D and 3D tracks are saved in comma-separated values (CSV) formats.

### Dataset preparation

Recordings of bumblebees from another experiment were used as training and validation data. The original experimental setup consists of a plexiglass tunnel of 2000*mm* length and a cross-section of 300*x*300*mm*^2^. This tunnel is connected to the hive of the bumblebees. The bees must fly through the tunnel before accessing the outer world. The tunnel was filled with one rectangular obstacle at a time. Obstacles of different sizes were available, leading to various changes in background and lighting in the tunnel. Three cameras filmed the experimental area in the tunnel from different angles for flight behavior investigation. Videos were recorded on different days and times of the day with different light conditions. A detailed discussion on the test setup can be found in [36].

**Fig 3:**
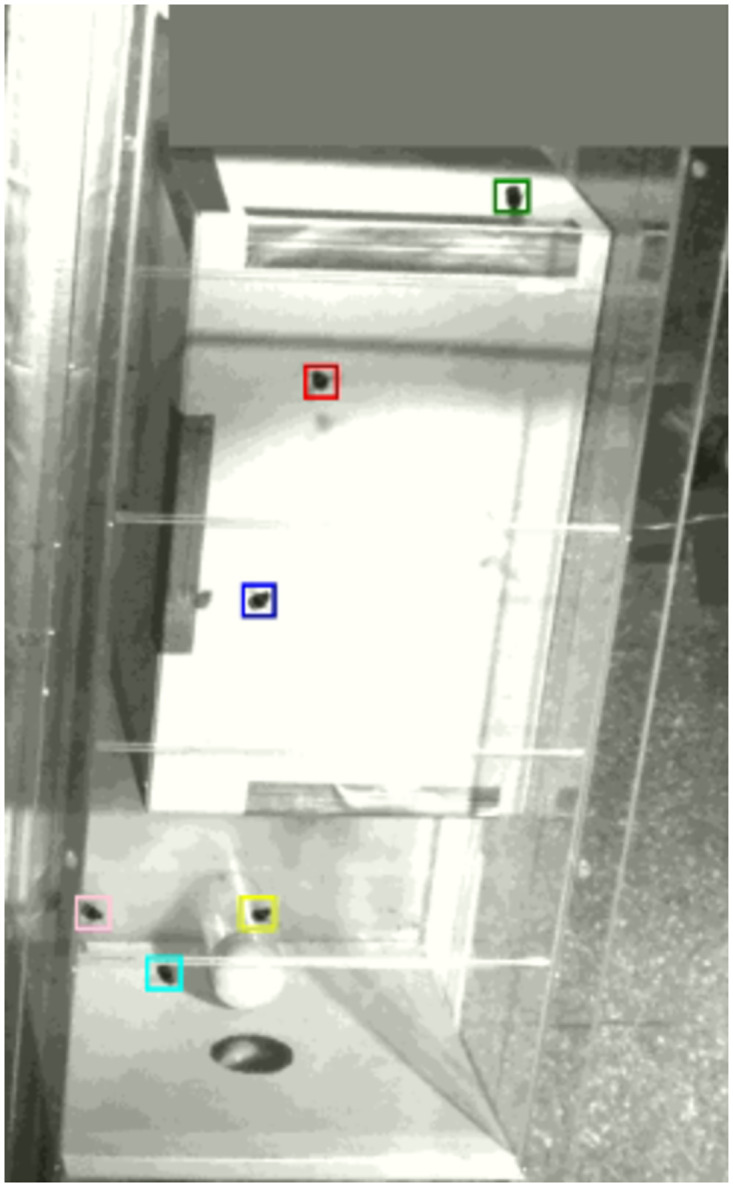
Example of labeled image for the bumblebee dataset for detector training.

Our work provides an open training dataset for *Bombus terrestris*. The dataset for detector training contains 11359 training images with 15624 bounding box annotations and 1323 images for validation with 3447 bounding box annotations. The images have a size of 1696*x*1710 pixels. The annotations are defined as rectangular bounding boxes. The origin of the (*x*, *y*) is the top left corner, and (*w*, *h*) is the width and height of the bounding boxes. Furthermore, the coordinates *x*, *y*, *w*, *h* are normalized by image width and height. An example of a labeled image is shown in Fig 3. Dead and blurry bumblebees are greyed out to prevent the network from learning undesired features.

**Fig 4:**
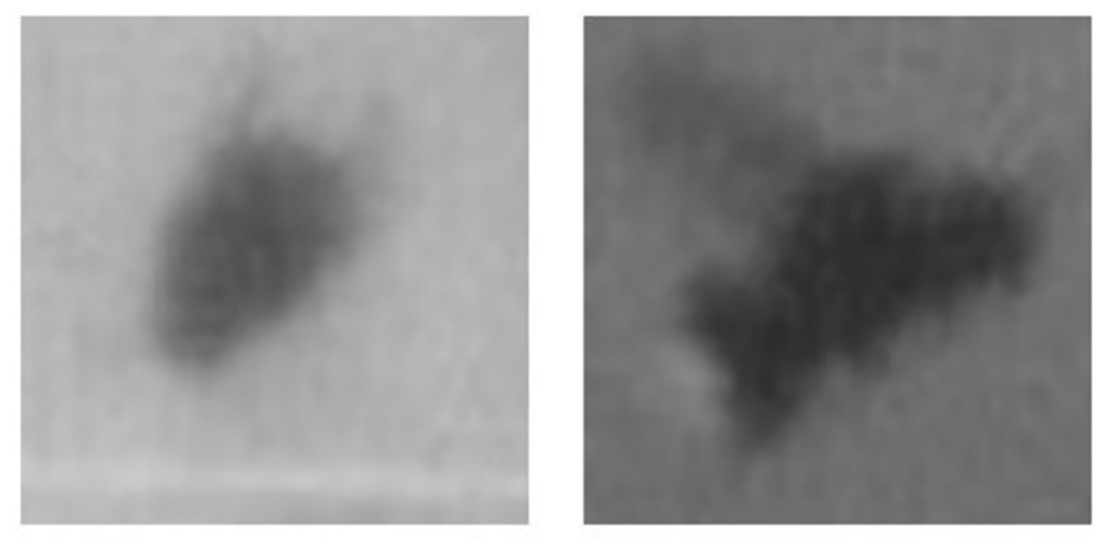
Training sample for appearance matcher.

The authors of the Multi-Ant Tracker [7] train the appearance match model on a small dataset containing 50 images. Initially, six ants were randomly selected with 8−10 random images, cropped to 95*x*95 pixels, to construct this dataset. While the ants were initially tracked from one camera angle only (from above) [7], here, the bumblebees must be tracked from the side view (camera 3) and top view (camera 1 and 2). Therefore, the bumblebee training dataset consists of twelve randomly selected bumblebees; six from the side-view and six from the top-view. 12 images were selected intentionally for each bee to represent every state of a wingbeat. They are cropped to 40*x*40 pixels and then upscaled to 95*x*95 pixels, the size envisaged by [7]. A Fast Super-Resolution Convolutional Neural Network (FSRCNN) implemented in the Python library OpenCV upscales the images. A training sample consists of two randomly selected, upscaled crops, as exemplarily shown in Fig 4.

We labeled four videos with 500 images each, with bounding boxes and the corresponding bee identities for validation. Two videos are from the side view angle, showing 2–5 bees simultaneously. The other two videos are from the top view, showing 2–4 bees simultaneously.

## Results

### Training detector

**Fig 5:**
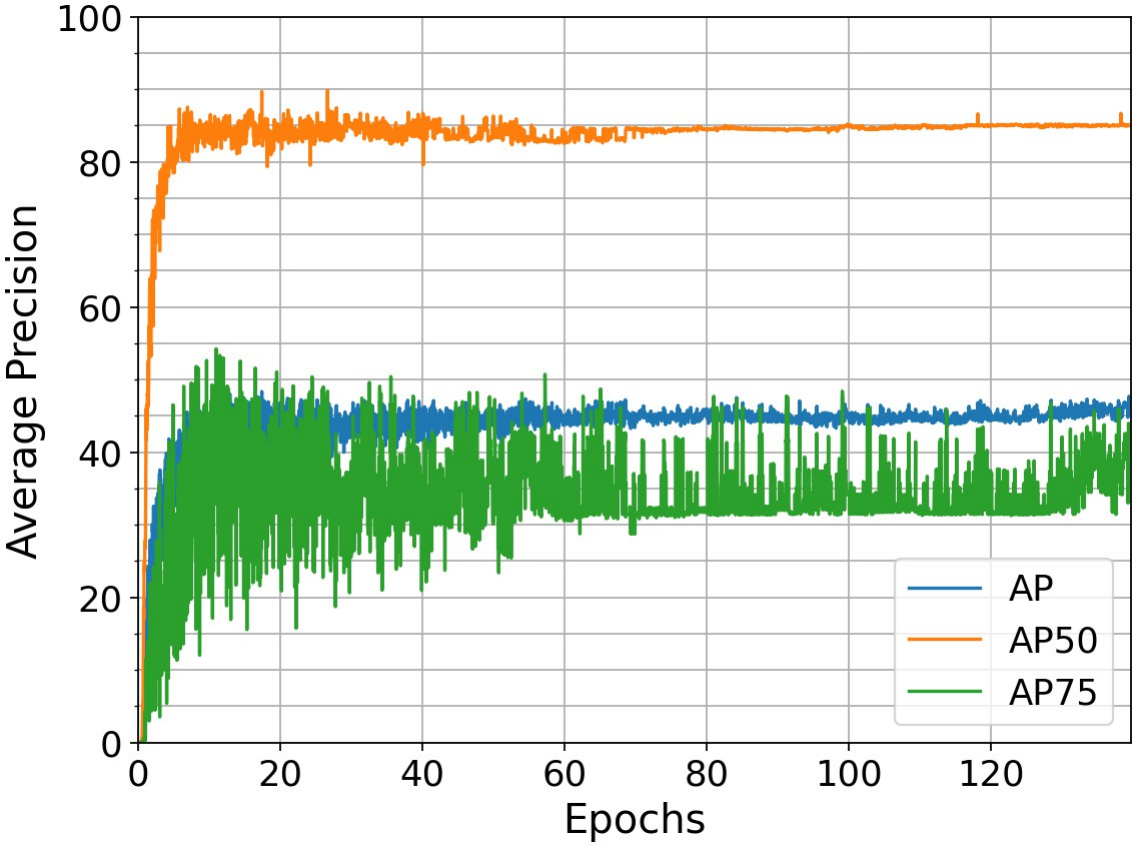
*AP*(50: 95), *AP*50 and *AP*75 (on the validation dataset) over epochs for Faster R-CNN, minibatch size 16, *anchor*_*sizes* ⋅ 0.75. After 60 epochs, an *AP*50 = 83, 8%, *AP*75 = 34, 2% and *AP* = 45, 3% is achieved.

The Detectors RetinaNet and Faster R-CNN training was conducted using the Detectron2 API [37] developed by Facebook Research. Facebook Research provides both models pre-trained on the Common Objects in Context (COCO) dataset [38]. The COCO trained Faster R-CNN model is used to finetune the model on the bumblebee dataset. The training results are plotted in Fig 5. The first two layers of the backbone are frozen. According to [39], reducing the anchor sizes can improve the learning of a detector for small object detections such as insects. By default, Detectron2 uses *anchor*_*sizes* = [32, 64, 128, 256, 512] (area of anchors in pixel square) for Faster R-CNN’s region proposal network. Several modifications were tested, such as *anchor*_*size* reductions by 25%, 50%, 75%. The total number of anchors increases with decreasing size to obtain the average overlap between them. Reducing the *anchor*_*sizes* by 25% leads to minor improvements. The minibatch size is the number of images fed to the network per training iteration, which is sixteen. Training starts with a learning rate of 5⋅10^−5^ after a warm-up over the first 1000 iterations. A learning rate schedule is introduced, decaying the learning rate at 28, 42, 55, and 70 epochs by the factor 0.5. An epoch is a full training cycle with all training data fed to the network exactly once, i.e., the sum of all batches. To prevent overfitting, training is stopped at 60 epochs achieving *AP*50 = 83, 8%, *AP*75 = 34, 2%, and *AP* = 45, 3%.

Here, we adopt the definition of *AP* by COCO [38]. *AP* is averaged Precision $P=\frac{tp}{tp+fp}$ over the Intersection over Union (*IoU*) range 0.5: 0.05: 0.95, considering the 100 top-scoring detections per image.

**Fig 6:**
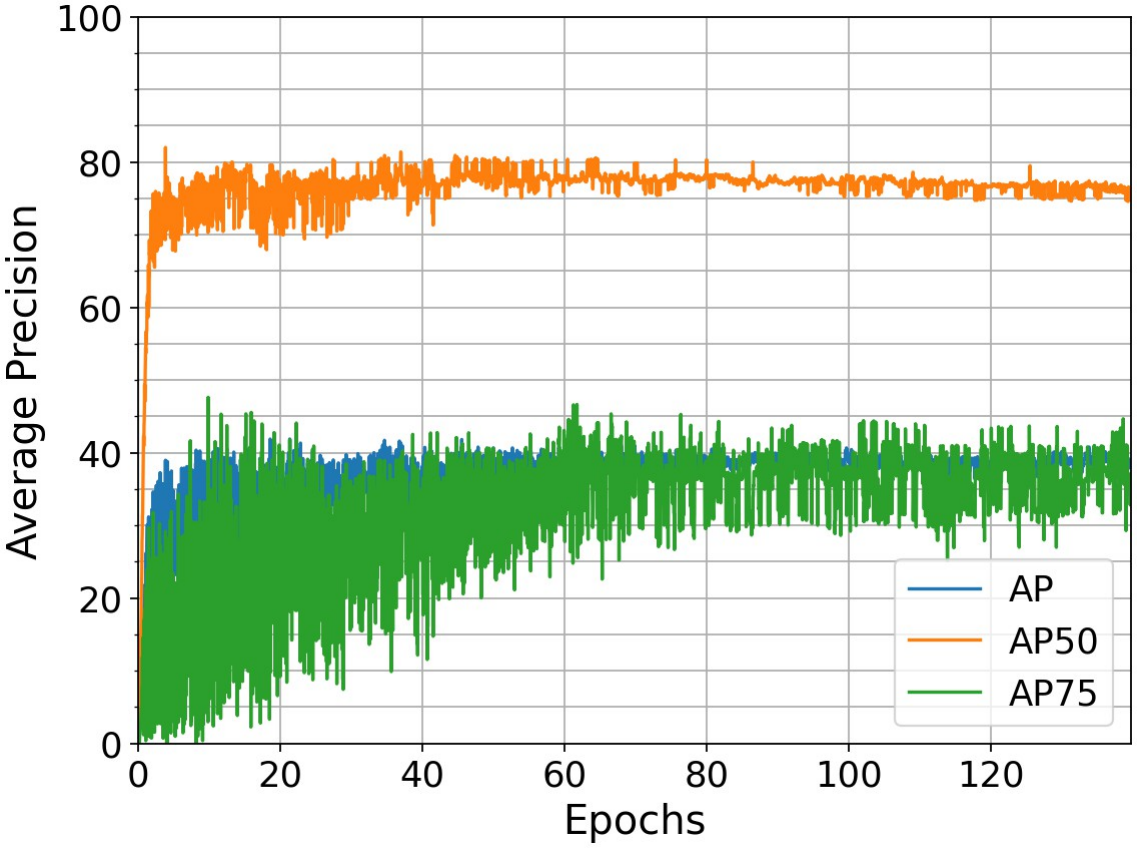
*AP*(50: 95), *AP*50 and *AP*75 (on the validation dataset) over epochs for RetinaNet, minibatch size 16. After 80 epochs, an *AP*50 = 77, 6%, *AP*75 = 37, 0% and *AP* = 38, 4% is achieved.

Training the RetinaNet is similar to the procedure presented for the Faster R-CNN. The COCO-trained RetinaNet model is used to finetune the model on the bumblebee dataset for weight initialization. The default hyperparameter settings of the Detectron2 Default Trainer [37] are used unless otherwise stated. The first two layers of the backbone are frozen. The focal parameter is *γ* = 5. Similar to Faster R-CNN, a minibatch size of 16 performs the best. The learning rate schedule starts with 8⋅10^−5^ after a warm-up over the first 1000 iterations. At 28, 42, 55, and 70 epochs the learning rate halves. This training session delivers benchmarks of *AP*50 = 77, 6%, *AP*75 = 37, 0% and *AP* = 38, 4% at 80 epochs as shown in Fig 6.

**Fig 7:**
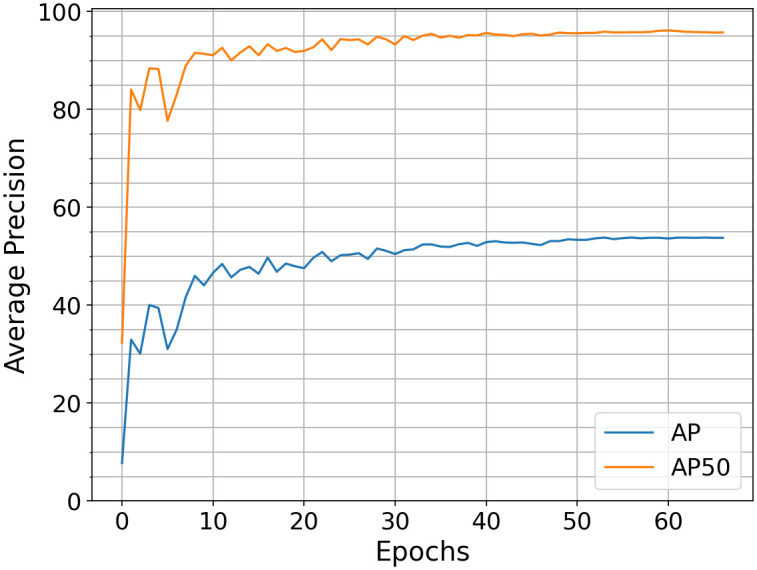
*AP*((50: 95)), *AP*50 and *AP*75 (on the validation dataset) over epochs for YOLOv5, minibatch size 16. After 60 epochs, an *AP*50 = 95, 8% and *AP* = 53, 8% is achieved.

YOLOv5 comes with an in-house training API used to train the COCO pre-trained YOLOv5l weights on the bumblebee class. We chose a minibatch size of 16, an image size of 640, and the multi-scale flag is True. According to [29], multi-scale randomly resizes the image by a factor -0.3:0.3. We kept all remaining hyperparameters on default, leading to the training session in Fig 7. Both benchmarks stagnate at approximately 60 epochs, which is the dropout point for the training, resulting in *AP*50 = 95, 8% and *AP* = 53, 8%.

### Training tracker

**Fig 8:**
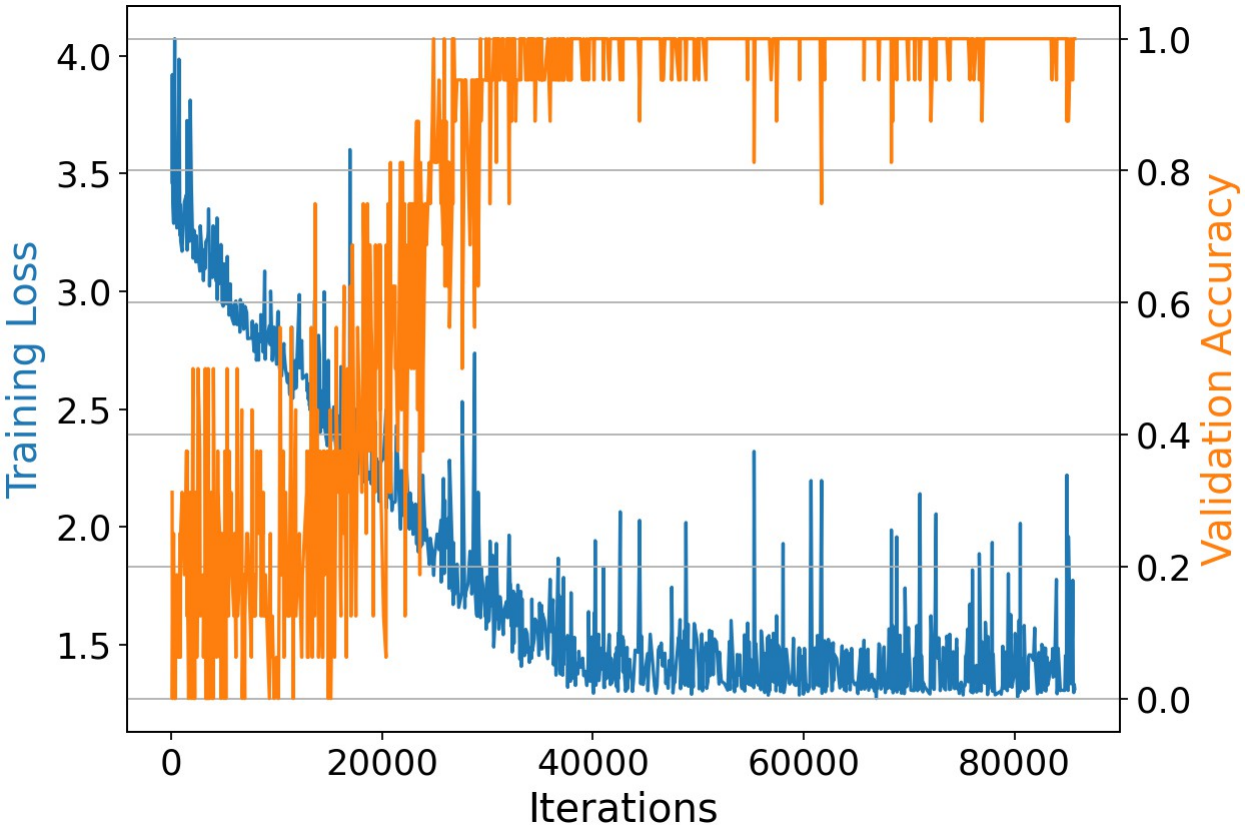
Training loss and validation accuracy for appearance match model of the MBT3D trained on bumblebee training dataset. After 40000 iterations, an accuracy of >99% is reached.

Training the appearance match model of the MBT3D was analog to [7], who published an API for training with suitable hyperparameters. Since the appearance matcher is a shallow network, it can be trained from scratch, using the truncated normal distribution for random weight initialization. The plot of the training session is shown in Fig 8. Training loss and validation accuracy reach their optimum after 40, 000 iterations and do not improve further; therefore, dropping out from training is valid here. The validation accuracy reaches >99%.

### Detector benchmarks

The final benchmarks of the three detector models are summarized in Table 1. RetinaNet has slightly worse performances in *AP*, *AP*50, and *AP*75 than the Faster R-CNN model. However, it is almost double as speed efficient as Faster R-CNN. YOLOv5 stands out in accuracy and speed with an impressive *AP* = 53, 8% while processing 62 frames per second. These benchmarks were calculated on 635*x*640 sized images. In order to perform predictions on the GPU, the images must be read from the SSD as NumPy arrays, transformed to GPU readable tensors, and sent to the GPU ram. After prediction, the GPU tensors convert to CPU NumPy arrays. This process is computationally expensive due to the high dimensions of the arrays. The Framerate is measured from the first moment of reading the image to the RAM until the model’s output converts to CPU NumPy arrays.

**Table 1:** Final detector benchmarks comparison on bumblebee dataset. The benchmarks were determined on an image size of 640.

	*AP*(50: 95)	*AP*50	*AP*75	*fps*
X101-FPN Faster R-CNN	45, 3%	83, 8%	34, 2%	14
R101 RetinaNet	38, 4%	77, 6%	37, 0%	24
YOLOv5l	**53, 8**%	**95, 9**%	−	**62**

### Tracker benchmarks

The following MOT challenge benchmarks [14] are used to evaluate the performance of the Tracker:

- False positives (*fp*): Total number of false positives
- False negatives (*fn*): Total number of false negatives
- Identity switches (*IDS*): Total number of Identity switches
- Fragments (*FM*): Total number of incidents where the tracking result interrupts the real trajectory
- Precision (*P*): ratio of $\frac{tp}{tp+fp}$
- Recall (*R*): ratio of $\frac{tp}{tp+fn}$
- MOT Accuracy (*MOTA*): combines the error sources *fp*, *fn* and *IDS*
- MOT Precision (*MOTP*): misalignment between annotated and predicted bound
- Frames per second (*fps*): measured for whole process detection, tracking and reconstruction

First, MBT3D is evaluated by performing data association on the validation dataset and using (hand-labeled) ground truth bounding boxes as input. For this setting, the tracker reaches a *MOTA* of 99, 4% and a *MOTP* of 94, 1% on the bumblebee dataset. For comparison, the Multi-Ant Tracker achieves a *MOTA* of 99, 3% and a *MOTP* of 91, 9% on the original ant dataset [7].

**Fig 9:**
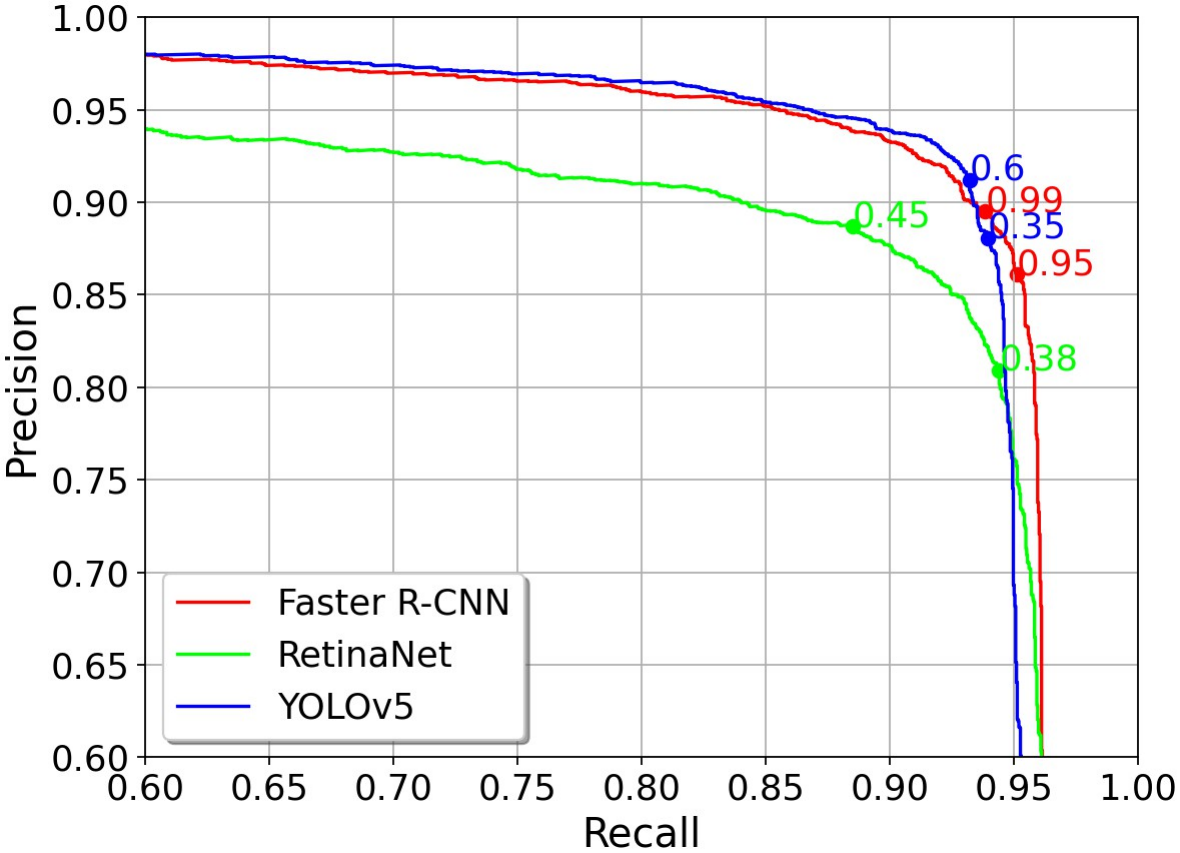
Precision over recall curves with a IoU threshold of 50% for the Faster R-CNN (red), Retina (green) and YOLOv5 (blue) models trained on bumblebee validation dataset. The dots denote the precision and recall for a specific confidence threshold. The higher the confidence threshold, the higher the precision, but the lower the recall, and vice versa.

Second, the various detection models feed their bounding boxes to the tracker, which identifies the individual tracks. The detection models require a suitable confidence score threshold (*CT*) to do so. The confidence threshold influences precision and recall of the detector’s predictions. Thus, setting the *CT* is a trade-off between these parameters. Depending on the detector’s task, precision or recall is crucial. In the given task, the detector is supposed to deliver robust detections for the Multi-Ant Tracker. The tracker recovers better from false negative detections *fn* than from false positives *fp*. MBT3D maintains the objects’ appearance over 100 frames to recover from object occlusions. For the tracker, a false negative during detection is similar to occlusion, and therefore, the tracker should be able to recover from it. A false positive might confuse the tracker because it either matches this false positive with a consisting track or initializes a new track. Therefore, precision is more important than recall to solve the given problem. Fig 9 shows the precision over recall curve for the Faster R-CNN (red curve), RetinaNet (green curve), and the YOLOv5 model (blue curve) using an *IoU* threshold of 50% on the bumblebee validation dataset.

For Faster R-CNN, confidence scores below 95% (red dot) decrease precision values while recall barely improves. For higher confidence scores than 95%, precision still improves while the recall drops drastically. Given that, confidence thresholds around 95%±5% were tested on the validation dataset. For the Faster R-CNN detector, the tracker performed best with a *CT* of 99%. An analog procedure was conducted for the RetinaNet and YOLOv5 model, determining a suitable *CT* of 45% (green dot) and 35% (blue dot), respectively. The benchmarks of MBT3D are listed in Table 2. MBT3D(Faster R-CNN) reaches top performances with *FM* = 44, *R* = 95, 5%, *MOTA* = 93, 5% and *MOTP* = 75, 6%. MBT3D(YOLOv5l) defines top benchmarks in *fp* = 89 and *P* = 98, 2% while running at 26Â *fps*. The *fps* is measured for detection, appearance description, and data association for the tracking process. Both MBT3D(YOLOv5l) and MBT3D(Faster R-CNN) show 5Â *IDS* on the validation set.

**Table 2:** Final MBT3D benchmarks comparison on bumblebee dataset. The benchmarks were determined on an image size of 640. The table shows the performance of the tracker using the upstream detections of X101-FPN Faster R-CNN, R101 RetinaNet and YOLOv5l model.

	*fp*↓	*fn*↓	*IDS*↓	*FM*↓	*P*↑	*R*↑	*MOTA*↑	*MOTP*↑	*fps*↑
MBT3D(X101-FPN Faster R-CNN)	98	**235**	**5**	**44**	98, 1%	**95, 5**%	**93, 5**%	**75, 6**%	11
MBT3D(R101 RetinaNet)	144	415	15	79	97, 1%	92%	88, 9%	72, 0%	16
MBT3D(YOLOv5l)	**89**	344	**5**	46	**98, 2**%	93, 4%	91, 5%	74, 4%	**26**

### Reconstruction

The 3D reconstruction is performed by two camera pairs independently, camera pairs 12 and 13. The used algorithm is based on triangulation. The center of each camera lens is the origin of its Coordinate Systems (COS), with *z* pointing in the focal direction. The coordinate system of camera 1 also serves as the global coordinate system.

**Fig 10:**
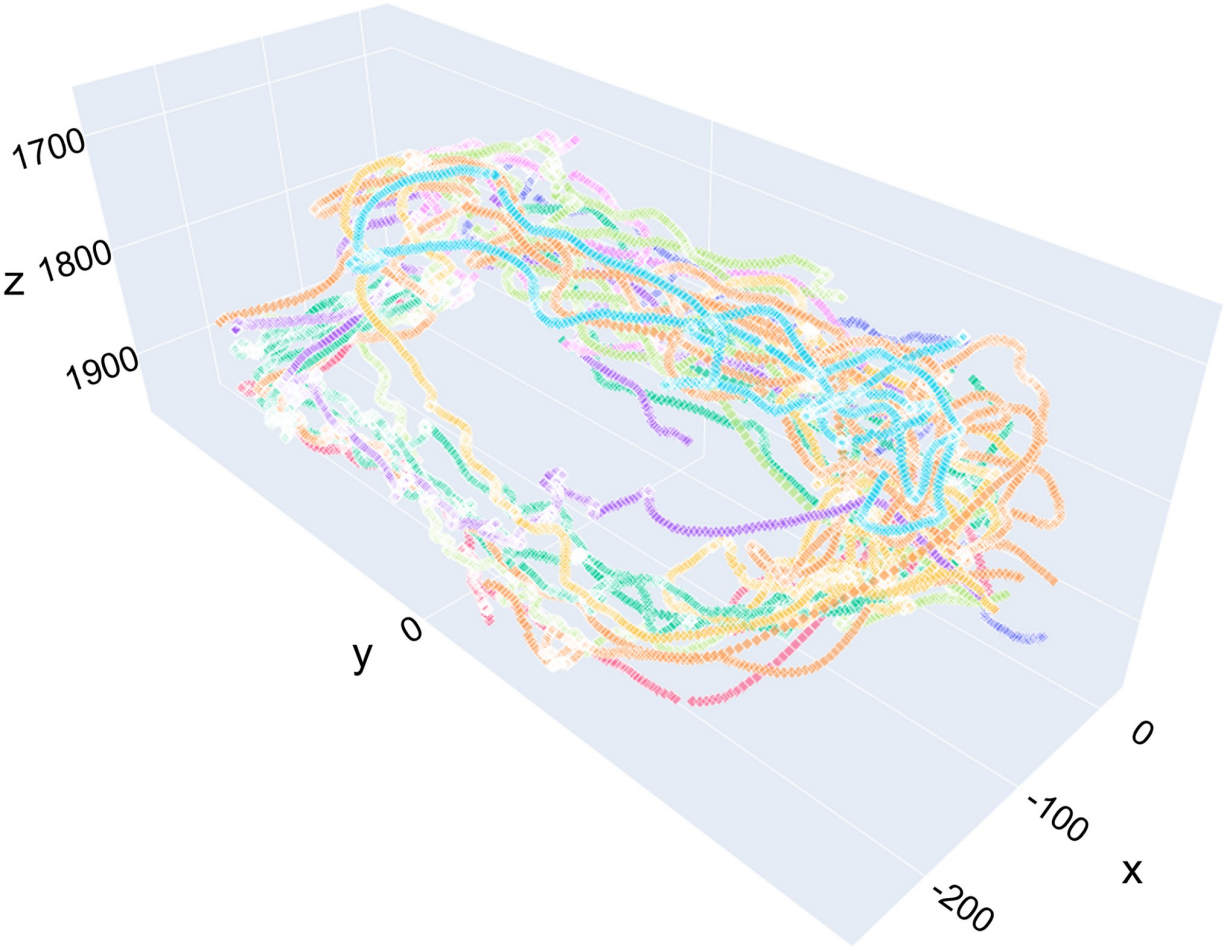
Example sequence for 3*D* path estimation with multiple bee identities. A unique color is assigned to every bumblebee identity. The shown paths were reconstructed using camera pair 12.

Fig 10 shows the 3*D* path estimations by camera pair 12 of an arbitrary sequence with around 5000 images and up to 10 bee identities simultaneously visible. Every bumblebee identity has a unique color. As demonstrated, the 2*D* tracks are successfully matched by the reconstructor algorithm 1, and the tracker reliably identifies individuals in multi-object sequences, despite the high complexity and intricacy of the trajectories.

## Performance compared to other trackers

This chapter compares the tracking tool introduced in this work with several tools commonly used in the ethology and biology community. Again, four challenging videos with 500 frames each serve as test videos. All four videos show the same experimental set-up, consisting of a perspex tunnel with large cuboidal obstacles. Two videos were filmed from the side and two from above. One video per position was taken in the morning and one in the afternoon, representing different lighting conditions. The lighting is non-uniform, and the obstacle has a big shadow. The two cameras used to film the videos have different distances to the test area. Therefore, bumblebees appear in different sizes between videos. All videos contain multiple bumblebees, *Bombus terrestris*. In some videos, dead bumblebees lie inside the test area. We tested four different tracking tools commonly used in the ethology and biology community. Every tool was initially developed for a slightly different purpose and has its own requirements. We used the tools as they were intended to achieve the best possible performance.

### Blob approaches

One of the first and most commonly used approaches in animal detection is identifying animals by defining an image’s foreground and background. The foreground pixels, i.e., those different from the background at a given position in the image, are clustered into spatially connected groups. Expectation-Maximization commonly does this grouping, e.g., in [40]. This approach, in its simplest form, has several disadvantages. First, not the animal itself is tracked but any difference between foreground and background. However, this difference might have various sources, e.g., the animal’s shadow, and lead to wrong detections. Additionally, partly occluding or touching animals are not separated anymore, as they form one connected “blob” of pixels. Some approaches try to solve this problem by taking the appearance into account (e.g., [41]) or suggesting boundary locations (e.g., in [42]). Another approach is predicting animal position by its movement, assuming that animals move slowly between images and only minor changes occur (e.g., in [43]). [44] use CNNs to determine the number of animals per blob and assign them identities.

However, non of this addresses the basic problem that not the animal itself is detected but differences between images. Even though several measures are taken to refine the results of the initial blob approach, this approach is prone to many false positives. The authors tested several blob-based approaches, including one self-developed one. The performance of these approaches differed quite significantly. The best blob-based trackers are discussed in the following sub-chapters.

#### Ctrax

Ctrax was published by [45] to automatically quantify individual and social behaviors of *Drosophila melanogaster* in a planar arena. First, the algorithm tracks the movement of individuals. Second, a classification algorithm differentiates several behaviors of individuals and creates an ethogram representing the behavior. The tracking task splits up into detection and identity assignment. The algorithm detects all individuals in every frame based on background subtraction. The pixel-wise median of a set of frames from the entire video sequence gives the background. The pixel-wise median absolute deviation from the background image gives the variability. A pixel is in the foreground if the intensity difference between pixel and background is higher than a multiple of the background variability. A group of connected foreground pixels lying inside an ellipse gives one identity. Identities are split or merged if they are too small or too large. The identity assignment is based on a constant velocity model, which predicts the position of an individual in the current frame based on its position in the previous frames. The detected positions of all individuals are compared to the estimated positions simultaneously. The Hungarian method for minimum-weight perfect bipartite matching [46, 47] gives the best match of detected positions and estimated ones.

The algorithm struggled with several challenges of our test setup. Shadows and reflections of bees were frequently detected as individuals. Additionally, most identity switches occur between a bee and its reflection or shadow. Additionally, the algorithm could not reliably distinguish between two bees that fly over each other and summed them up as one identity. The algorithm also assigned two identities to the same bee multiple times. This multi-assignment usually happens when the white part of the bee is similar to the background. Then the dark tail and the dark head are identified as individual identities. This error often propagates for many frames. This observation also fits a statement of Branson et al. in their initial work, who state that their approach fails if flies look too similar to the background [45].

#### idTracker.es

idTracker.es is a multi-tracking algorithm that extracts a characteristic fingerprint for each animal in a video stream [40]. The fingerprint is then used to identify the animals in the frames of the video stream. idTracker.es uses the idea of color correlograms of the image but simplifies the color correlogram to a 2D representation. A representation of the distance *d* of every pair of pixels *i*_1_ and *i*_2_ of the segmented animal is formed. The intensity map is a histogram of the representation *d* vs. *i*_1_ + *i*_2_. The contrast map is a histogram of the representation *d* vs. |*i*_1_ − *i*_2_|. These two histograms form the fingerprint of the animal. idTracker.es accounts for differences in the posture of one animal by simply using a whole reference set with multiple postures to classify each animal. A simple blob approach performs segmentation. All connected pixels that lie above (or below) a user defined intensity threshold belong to one blob. Additionally, blobs that are smaller than a user-defined threshold are discarded.

We tested idTracker with our data set and achieved worse performance compared to the performance of the idTracker in its initial presentation in [40]. We tested all four of our testing videos with multiple intensity threshold settings and considered the best performance per video. We assume that an experienced user would know which threshold works best and chooses the correct threshold before starting the tracking. We also defined the region of interest only at the test area and performed the build-in background subtraction function before tracking. Despite our videos only being 17 seconds long, no ideal intensity threshold exists because all videos change in lighting conditions or have non-uniform lighting. However, the result was expected to a certain point. The initial publication and the user guide [48] state:

- Illumination should be uniform
- the size of individuals should not change
- objects which are only present in a part of the video are problematic

**Fig 11:**
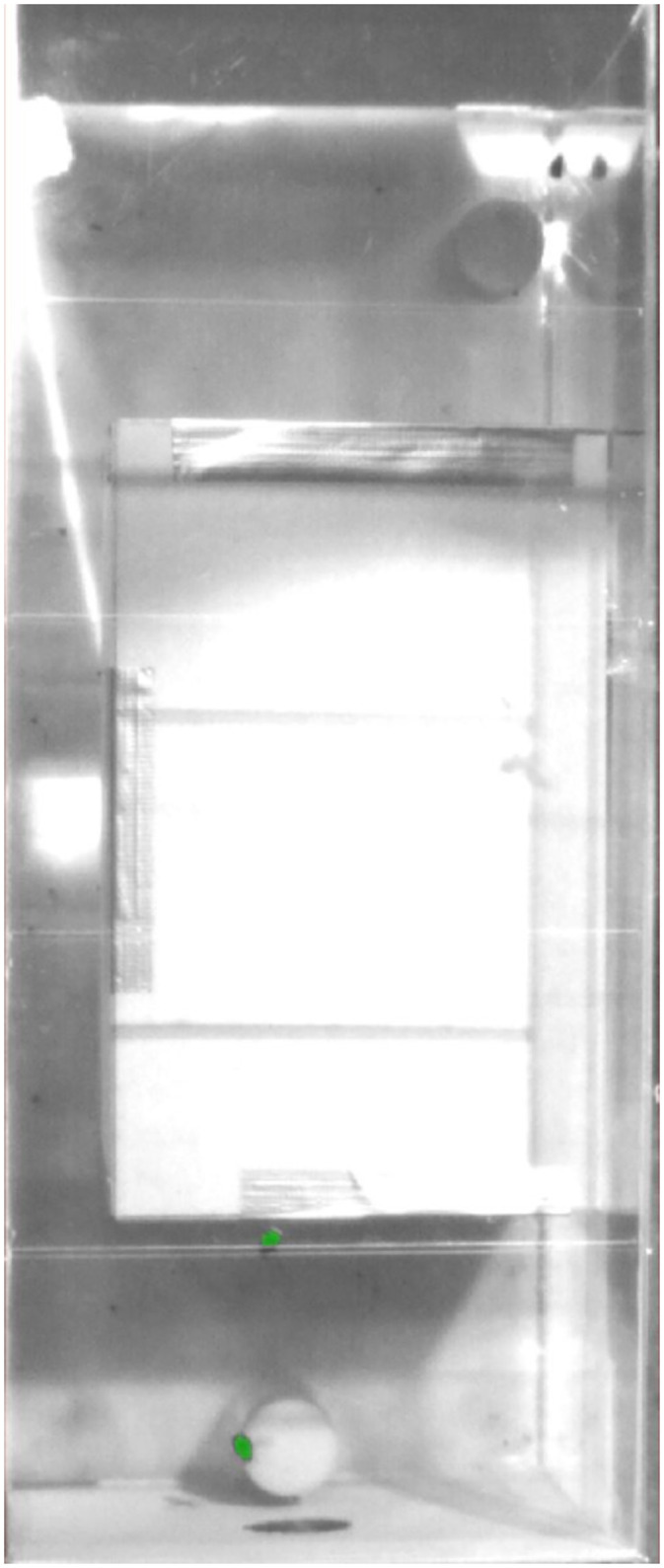
Example of idTracker.es tracking two bumblebees (bottom) while one bumblebee is not tracked (top).

The intensity threshold differentiating foreground and background is constant during one video. Finding an intensity threshold that worked well in all test section areas was impossible. Fig 11 shows an example in which the bumblebee on the top over bright background is not detected, whereas the others are detected. However, changing the intensity threshold such that all bees are detected in this frame leads to significantly more false positives in other frames. As all our videos have non-uniform illumination, we could not find an intensity threshold that reliably detected all bumblebees without many false positives.

Additionally, bees entered and left the test section during one video. Similar to the statement in the original publication, we found that this lead to additional false positives or negatives. Especially in the horizontal view the size of bumblebees changed quite significantly, when bumblebees turned 90°. Unlike the warning of the developers of the algorithm we did not find any indication that changes in size are problematic for the algorithm.

### Neural Network approaches

Recently Neural Networks have gained more and more popularity. They are well suited for various image analysis tasks. This chapter discusses tools and algorithms that use a Neural Network to detect an animal directly.

#### idTracker.ai

idTracker.ai is a software that extracts trajectories of large groups of up to 100 individuals [44]. It uses one CNN to detect when animals meet and one CNN for identity identification. Like idTracker.es, idTracker.ai uses a blob approach to define areas in each frame that correspond to one or more animals. Therefore, the detection task itself is primarily a simple blob approach. Then, the blobs are oriented according to their longest axis, and a CNN determines whether a blob contains only one or multiple (crossing or touching) animals. Then, another CNN identifies individual animals in all blobs with multiple animals. The first CNN, detecting the crossing or touching, is trained with a set of high confidence heuristic labels representing single or crossing animals. The second CNN, for identification, was initially trained with 3000 randomly selected images per animal and reached over 99, 9% accuracy. However, this approach proved impractical as this amount of images is not readily available for every experimental setup and individual. Therefore, the developers of idTracker.ai implemented three protocols. The first step of these protocols is the identification of all intervals with separate animals only. The interval is extended for every individual animal after and before this particular animal has the previous and next collision. These extended intervals are called global fragments and are used for training. Depending on the performance of the trained network, the global fragments are sorted and used slightly differently to retrain the network in Protocols 2 and 3 [44].

The authors of idTracker.ai usually achieved accuracies of 99.96%±0.06% to 99.99%±0.01% for various test cases. However, they also state that their network reaches its limits with groups of 80 to 100 flies, but it still achieves accuracies of 99.5%. Two concerns of the algorithm are the size of the global fragments, which should be >30 images per animal, and the image quality with around 300 pixels per animal in the segmentation step recommended [44]. The idTracker.ai user guide also states: “For a good algorithm performance, there must be multiple parts in the video where the number of blobs detected (marked in red in the preview window) is equal to the number of animals indicated in this text box.” [49]. Additionally, uniform lighting conditions are also recommended for idTracker.ai.

**Fig 12:**
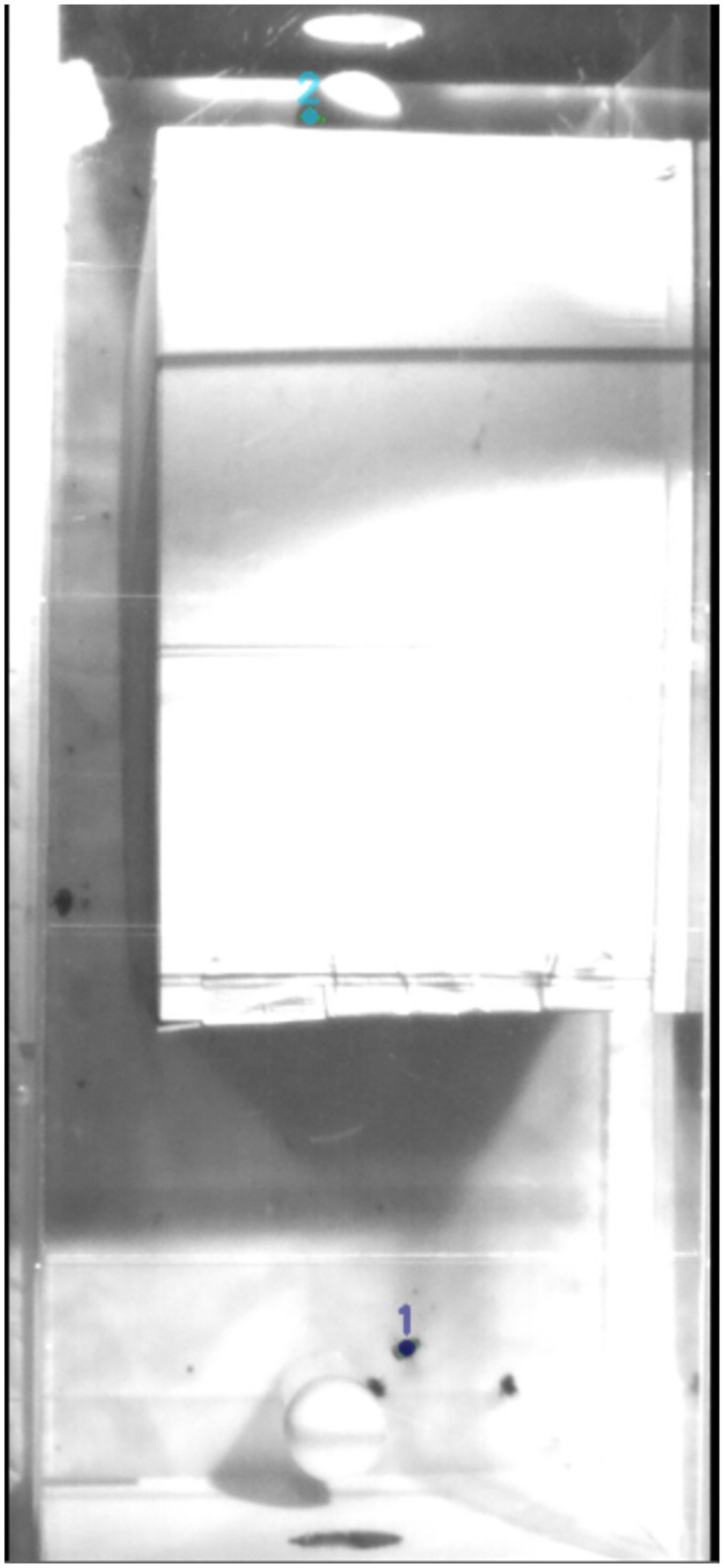
Example image of tracking in test video 2.

idTracker.ai has an easy-to-use Graphical User Interface (GUI). The intensity threshold (upper and lower limit) and the area threshold (upper and lower limit) are user-defined in this GUI. We tried several different settings and only considered the best-performing setting, assuming that an experienced user would know the ideal parameters. Nonetheless, we struggled to find parameters working well in all image sections. Fig 12 shows an example of such a situation. Bumblebee one is dark above bright background and identified. However, bumblebee two is currently flying in the shadow of the obstacle and, therefore, a dark blob above dark background. Instead of the actual bee, a random point, most likely a shadow, is detected in the upper part of the image as bee two. Please note that the two bees on the ground, next to bee two, are dead and not detected correctly.

#### DLTdv digitizing tool

Tyson Hedrick published his 3D animal tracking tool DLTdv3 in 2008 [50]. Currently, DLTdv8 is available on his website [51]. We review the main ideas of his initial publication and highlight some unpublished improvements of the recent version. DLTdv is a Matlab application with an easy-to-use user interface. DLTdv provides several functionalities for manual, semi-automatic, and fully-automatic tracking of 2D and 3D video streams. DLTdv allows tracking specific animal features, e.g., a wing tip or marking. DLTvd3 combines a two-dimensional cross-correlation tracking with several predictive tracking algorithms (Kalman and double exponential filters) and an object centroid refinement.

Besides the classical deterministic approach, DLTdv8 has a neural network integration. The neural network requires training to identify specific feature points. Training data can be generated in DLTdv8 by manually tracking some feature points in the video stream. The developer of DLTdv8 has not published a detailed description of the inner workings of the neural network. However, they rely on the Matlab standard procedure to train a network and use a CNN for image analysis. Therefore, Tyson Hedrick’s approach is close to the method presented in this work. However, he seems to rely on a pre-trained network that is refined for the given video stream. This approach allows learning new feature points very quickly. However, the overall network is less robust than ours. Our performance investigation also supports this.

DLTdv has several advantages. It allows for tracking arbitrary points with very low training effort. Additionally, the user interface is very clean and easy to follow, which gives users unfamiliar with programming and coding access to DLTdv.

We trained DLTdv8 with four approaches and present the two best working ones here. First, we trained the network as it is meant to be trained. We trained a new network for every video (i.e., camera position and daytime). We manually tracked one bumblebee in the video’s first 80 and last 20 frames. We used this data to train the network. Table 3 shows the performance of this approach as “DLTdv8 trained per video”. Second, we retrained the first network by manually tracking all bumblebees in the first 80 and the last 20 frames. While this slightly influenced false positives and negatives, the number of identity switches increased. The identity switched multiple times between close bumblebees. This behavior was prominent if an unknown bumblebee was in the video. Not all bumblebees were present in the 100 images for training. We assume that the tracker struggles with this unfamiliar identity causing many switches. Third, we trained one network by manually tracking one bumblebee in 50 images of all four video streams. Table 3 shows the results as “DLTdv8 trained once”. Finally, we trained the network with the same training data used for our network. We fed our training images and ground truth data adapted for DLTdv8 to the network. This training data consists of images from the same and additional camera positions, similar and different lighting conditions, and same and different obstacles and backgrounds. A network trained by this data performed the worst of all four tested variations. In the end, DLTdv8 is not designed to be trained on some arbitrary, completely different images teaching the network all possible appearances of a specific class. Nonetheless, the performance is astonishing if it is used the way it is meant to be used.

**Table 3:** Comparison of the performance of the proposed tool with other commonly used tools for animal tracking.

*Tool*	*IDS*↓	*fp*↓	*fn*↓
MBT3D(X101-FPN Faster R-CNN)	**5**	98	235
MBT3D(R101 RetinaNet)	15	144	415
MBT3D(YOLOv5l)	**5**	89	344
DLTdv8 trained per Video	21	**41**	**48**
DLTdv8 trained once	47	229	233
Ctrax	9	1409	59
idTracker.es	−	148	195
idTracker.ai	34	788	503

### Comparison

Table 3 shows the false positive, false negative, and identity switches for the different tools for four test videos with 500 frames each. Identity Switches (IDS) are considered the most critical criterion. IDS might lead to wrong conclusions or show wrong paths if an individual’s assignment changes. We consider false positives a second important criterion as false positives might disturb the whole tracking process or distort the analysis. Lastly, we consider false negatives as the least important criterion. As long as they only occur rarely, a feasible interpolation method should mitigate the effect of failed detection. MBT3D(X101-FPN Faster R-CNN) and MBT3D(YOLOv5l) proposed in this work have the lowest number of identity switches. DLTdv8 has the lowest false positives and false negatives if a network trained with the particular video it finishes to analyze is used.

Two of our approaches have the lowest number of identity switches, followed by Ctrax. This is not surprising as these algorithms were specifically designed to track multiple animals. idTracker.ai has the second-highest number of IDS, which is quite surprising as this algorithm was developed to track multiple animals and generally achieves very high accuracy. Most of idTracker.ais IDS occurred with false positives and very rarely between two bumblebees. The algorithm also gets confused when a new individual enters the test arena or leaves. idTracker.ai was primarily developed to identify and track a constant number of animals. Therefore, this algorithm has a great disadvantage in our test scenario. DLTdv8 trained with a general training data set has the highest number of IDS. Usually, the IDS occurred between very close bees. The appearance of the bumblebees is not different enough for DLTdv8 to reliably distinguish different individuals. The IDS are cut in half if DLTdv8 is trained separately for every video. If the network is trained with only one video, it learns to identify bumblebees from the side or the top, not both. This reduced learning complexity seems to increase the network’s ability to distinguish different individuals.

DLTdv8 has only 41 false positives if its network is trained with 100 frames of the video it is analyzing. However, in our tests, DLTdv8 stopped 19 times because the neural network could not identify one bee. One solution to this is reducing the required tracker confidence score further. However, a lower threshold led to more false positives. So we had to track the bumblebees in 19 frames manually. Our proposed tracker achieves the next lowest number of false positives without any manual assistance. MBT3D(YOLOv5l) and MBT3D(X101-FPN Faster R-CNN) have 89 and 98 false positives. However, both networks are trained on a general data set and not specifically for the chosen four test videos. The purely blob-based idTracker.es ranks fifth with 148 false positives. DLTdv8 performs much worse if it is trained only once and the same network is used for all four videos. In this case, DLTdv8 has 229 false positives. idTracker.ai has 788 false positives. Despite being the successor of idTracker.es and having a blob-based approach, we were unable to find a parameter setting in which idTracker.ai performs similarly to idTracker.es. Ctrax is too sensitive for our experimental set-up. It commonly detected shadows and reflections as bumblebees. Ctrax has a wrong detection in three out of four images.

DLTdv8 also achieves the lowest number of false negatives with 48. However, it only achieves this low number of false negatives if the network is trained explicitly for every video. If a more general network is used, false negatives increase to 233. This number of false negatives is similar to the performance of our tracker with Faster R-CNN, which has 235 false negatives. However, if our tracker uses RetinaNet or YOLOv5l, false negatives are higher. This is expected for our approach as we defined false positives as more critical than false negatives. For our approach, a false negative is the same as an occlusion. A false negative is mitigated by linear interpolation between the last and the new point. Therefore, false negatives do not yield any problem for our framework as long as they only occur occasionally. As expected, the blob approaches have a very low number of false negatives. However, this comes at the cost of many false positives. We could not significantly reduce the number of false positives of Ctrax, so we decided to minimize the number of false negatives. Here, idTracker.ai performs worst; surprising for a partly blob-based approach. The problem is the control parameters that define the blobs. The lighting of our experimental set-up is too inconsistent to find one good parameter setting. The authors of idTracker.ai emphasized that uniform lighting is important for their algorithm to work [44]. Therefore, this algorithm has a disadvantage again.

In summary, DLTdv8 is best for very specific tasks. The neural network’s easy training allows for reliably tracking arbitrary feature points. Unfortunately, easy and quick training only allows for superficial learning. The more variable the task is, e.g., tracking at different lighting conditions or experimental set-ups, the worse the performance. So different networks are required if anything in the experimental set-up changes. DLTdv8 has a detector coupled with a simple motion model, leading to identity switches if animals collide or are close. Contrary, our approach can identify a particular individual much better than the other algorithms. It can also handle variabilities better and has lower requirements on the experimental set-up (e.g., uniform lighting, no changing conditions). Uncertainties in the video, e.g., a person partly walking in the video or other kinds of movement, do not disturb our tracking. Our framework could also easily be extended to differentiate between two species to investigate their interaction. If large numbers of videos need to be processed our approach proved to be best suited, especially with challenging lighting conditions and multiple, interacting individuals.

## Conclusion

We proposed a method for 3*D* flight path estimations of bumblebee video sequences. An offline trained appearance descriptor paired with a Kalman Filter associates detections provided by an arbitrary detector model. The experimental results demonstrate that the novel method is robust regarding identity switches, fragments, and long-term occlusions. The MBT3D(ground truth) reaches a *MOTA* of 99, 4% and a *MOTP* of 94, 1% (association on ground truth bounding boxes) on the bumblebee dataset. The original Multi-Ant Tracker [7] achieves a *MOTA* of 99, 3% and a *MOTP* of 91, 9% on the ants dataset. Thus, the proposed method reaches state-of-the-art Computer Vision benchmarks. The proposed detection models were successfully trained on the bumblebee dataset, especially the YOLOv5l model delivers remarkable benchmarks (e.g., *AP* = 53, 8%) with an impressive speed of 62Â *fps*. However, combining the detection models with the Multi-Ant Tracker, the MBT3D(X101-FPN Faster R-CNN) surpasses the MBT3D(YOLOv5l) in most of the tracking benchmarks. Our approach has the least Identity Switches compared to other commonly used algorithms in biology and ethology. It can also best handle changes in the experimental set-up, lighting conditions, or camera position. In general, our approach is robust against various changes in the video. It proved to track bumblebees in various situations without input from a human operator. However, our approach is only able to track full bumblebees. Contrary, DLTdv8 is so easy to train that new conditions can be taken care of by retraining (or new training) of its network. If DLTdv8 is adjusted to new conditions, it has fewer false positives and negatives than our approach. However, in our test cases, it is still prone to IDS and needs more fine-tuning by an operator than our approach.

In conclusion, while our proposed framework shows promising results in tracking bumblebees, there are some limitations that need to be addressed in future work. One important aspect to consider is addressing the challenge of tracking half-visible bumblebees, which requires expanding the training dataset with image samples specifically addressing this scenario. Another limitation to acknowledge is that the current dataset was created under controlled laboratory conditions, which may lead to slight model overfitting and potentially lower performance in real-world conditions. To mitigate this, future work should focus on extending the dataset to include bumblebee images captured in their natural environment. By addressing these limitations and incorporating these improvements, the framework can be further refined to enhance its tracking capabilities in more complex and diverse scenarios.

## Supporting information

S1 Video Tracking video example 1. (MP4)

S2 Video Tracking video example 2. (MP4)

S1 Plot 3D path example 1, camera pair 12. (HTML)

S2 Plot 3D path example 2, camera pair 12. (HTML)
